# An algorithmic approach to determine expertise development using object-related gaze pattern sequences

**DOI:** 10.3758/s13428-021-01652-z

**Published:** 2021-07-13

**Authors:** Felix S. Wang, Céline Gianduzzo, Mirko Meboldt, Quentin Lohmeyer

**Affiliations:** ETH Zurich, Leonhardstrasse 21, 8092 Zurich, Switzerland

**Keywords:** Eye-tracking, Expertise development, Gaze patterns, Novice training, Sequence analysis

## Abstract

Eye tracking (ET) technology is increasingly utilized to quantify visual behavior in the study of the development of domain-specific expertise. However, the identification and measurement of distinct gaze patterns using traditional ET metrics has been challenging, and the insights gained shown to be inconclusive about the nature of expert gaze behavior. In this article, we introduce an algorithmic approach for the extraction of object-related gaze sequences and determine task-related expertise by investigating the development of gaze sequence patterns during a multi-trial study of a simplified airplane assembly task. We demonstrate the algorithm in a study where novice (*n* = 28) and expert (*n* = 2) eye movements were recorded in successive trials (*n* = 8), allowing us to verify whether similar patterns develop with increasing expertise. In the proposed approach, AOI sequences were transformed to string representation and processed using the *k*-mer method, a well-known method from the field of computational biology. Our results for expertise development suggest that basic tendencies are visible in traditional ET metrics, such as the *fixation duration,* but are much more evident for *k*-mers of *k* > 2. With increased on-task experience, the appearance of expert *k*-mer patterns in novice gaze sequences was shown to increase significantly (*p* < 0.001). The results illustrate that the multi-trial *k*-mer approach is suitable for revealing specific cognitive processes and can quantify learning progress using gaze patterns that include both spatial and temporal information, which could provide a valuable tool for novice training and expert assessment.

## Introduction

Advances in the technology of eye tracking (ET) have provided us with a deeper understanding of specific cognitive processes and the development of perceptual expertise. Using traditional ET metrics, the majority of studies have focused on the analysis of visual expertise by investigating the eye movements of individuals in a wide range of domains, such as teaching (McIntyre & Foulsham, 2018a), medicine (Castner et al., 2020; Fox & Faulkne-Jones, 2017; van der Gijp et al., 2017) or aviation (Haslbeck & Zhang, 2017). Traditionally, these studies use ET as summary statistics, such as fixation duration, dwell time duration, fixation count, or time to first fixation to analyze expertise specific gaze behavior (Cristino et al., 2010; Kanan et al., 2015; Ooms et al., 2012). These metrics, however, only capture the simplest of temporal information, while neglecting the temporal and spatial context of eye movements (Cristino et al., 2010). Studies using summary statistics have often generalized their findings, claiming that experts experience fewer fixations and shorter fixation durations, even though it has been established that expertise is highly domain-specific (Chi, 2006). Jarodzka and Boshuizen (2017) concluded that the reported measures are too reductionist to capture interesting insights into the nature of task- and stimuli-specific expert behaviors.

Researchers have since looked increasingly beyond traditional ET measures and have studied expertise by including the sequential information of gaze sequences. Sequential analysis has been shown to reveal a learner’s overall learning behavior (Tsai et al., 2012) and that in-depth analysis of individuals’ cognitive strategies during goal-oriented tasks (Hou et al., 2009) can be achieved. Moreover, Day et al. (2018) found that studies on expert and novice gaze behavior could benefit from identifying patterns that appear with a higher likelihood in a specific expertise group. Consequently, the comparison of gaze sequences using measures of string similarity, such as the Levensthein distance (Levenshtein, 1966), has successfully advanced our understanding of expertise development by showing that experts’ eye movements are more similar to each other than to those of lower expertise levels (Kanan et al., 2015; Tien et al., 2015; Watalingam et al., 2017). In these so-called *string-edit* approaches, such as ScanMatch (Cristino et al., 2010), a gaze sequence is converted into a string of letters, where each letter is assigned to a fixation onto a specific object or area of interest (AOI), thus conserving the temporal and spatial fixation information. Two sequences are then compared by counting the number of operations that are needed to convert one sequence into the other, which provides the similarity score (Anderson, Anderson, Kingstone, & Bischof, 2015a).

However, for the analysis of perceptual expertise development, two central aspects have yet to be considered. First, although the commonly applied string-edit approaches consider sequential gaze information during sequence comparison, the spatial and the temporal object relationship is lost after the similarity score calculation (Cristino et al., 2010; Day et al., 2018). Thus, after showing that differences between experts and novices exist, the question remains as to which task-specific gaze sequences can be consistently measured in expert eye movements and whether a suitable ET metric can be found that allows quantification of visual expertise development. Second, in the overwhelming majority of studies that investigate the development of expert gaze behavior, it is common practice that a single dataset of participants from different stages of development is recorded and that the gaze sequences of each group are then compared to specify expertise-related similarities (Castner et al., 2018; Eivazi et al., 2012). While this approach allows the analysis of how experts’ and novices’ eye movements differ at the specific time of the study, it stems from the assumption that all novices will eventually develop gaze sequences that are very similar to those of experts, with increasing training. However, to be able to truly reveal which behaviors are developed during training, multiple measurements on the same participants should be considered. Only then can we understand what behaviors drive the development of expert behavior within an individual under stable learning conditions (Gegenfurtner, 2013). Because of these limitations, a different approach to analyze gaze sequences is needed that can on one hand reveal task-specific gaze patterns and, on the other, allow the quantification of the development of expertise over time. In the field of computational biology, algorithms for quantitative DNA and protein sequence comparison approaches have been developed and optimized for decades. One well-established approach is *k*-mer analysis, which counts the frequency of subsequence patterns of neighboring elements with length *k* (2-mer, 3-mer, 4-mer, etc.) (Manekar & Sathe, 2018). The comparison of subsequences preserves the information of the composition of said sequences, which, in this case, is the sequence of letters representing fixations on AOIs.

In this article, we demonstrate the ability of a *k*-mer pattern approach to determine the development of expertise, by applying the algorithm to object-related gaze data of an ET study involving a natural handling task. By analyzing the sub-pattern frequencies over multiple successive trials of the same expert and novice subjects, we show that *k*-mer patterns can provide a suitable metric to quantify the development of task-related expertise.

## Related works

This section aims to highlight the previously conducted research in the field of visual expertise and sequence comparison approaches, and provides an overview of the *k*-mer analysis approach.

### Eye movements in the study of visual expertise

Eye tracking (ET) has established itself as a popular research tool for the study of behavioral patterns (Duchowski, 2017; Land & Hayhoe, 2001) and, due to easier accessibility of the technology, has been increasingly applied to investigate visual expertise and expertise development (Brunyé et al., 2014; Crowe et al., 2018; Kelly et al., 2016). Particularly in the field of medicine, it has been of increasing interest what constitutes expertise, to increase the effectiveness and efficiency of novice training and diagnostic accuracy (van der Gijp et al., 2017). Using ET summary statistics, Wood et al. (2013) found that during the interpretation of skeletal radiographs, experts when compared to novices exhibit shorter fixation durations and are faster to fixate on the site of the fracture. In a study on laparoscopic skill acquisition, Wilson et al. (2011) discovered that experts show more fixations on task-relevant areas, while Gegenfurtner et al. (2011) showed that experts have longer saccades and, again, shorter time to fixate on task-relevant information. Zimmermann et al. (2020) measured fewer AOI transitions between task-critical objects during expert trials compared to novices during a cardiovascular intervention. Conversely, other studies have reported that novices, not experts, focused more of their attention on the surgical task (Zheng et al., 2011) and that experts visited fewer task-relevant areas (Jaarsma et al., 2015). In their review on ET study results for visual diagnostic performance in radiology, van der Gijp et al. (2017) found conflicting results regarding the relationship between the level of expertise and ET summary statistics. While in all studies the number of fixations seems to decrease with high levels of expertise, no generalization could be made on AOI fixation durations, the number of fixations on AOIs, dwell time ratios, saccade lengths, or image coverage.

Van der Gijp’s results coincide with our knowledge that expertise is highly domain-specific (Beck et al., 2013; Chi, 2006; Sheridan & Reingold, 2017) and that results based on traditional ET summary statistics cannot and should not be generalized (Fox & Faulkne, 2017; Jarodzka & Boshuizen, 2017). Hence, in order to reveal more in-depth insights into the nature of perceptual expertise development, we are faced with the challenge of finding eye movement-based metrics that can help uncover task-specific, behavior-based development of expertise, while being generally applicable to a wide range of domains.

### String-edit approaches

First introduced by Noton and Stark (1971), the scanpath theory postulates that fixed viewing sequences are generated top-down as a result of the specific model of a subject. Using a string editing approach, Privitera and Stark (2000) were the first to achieve scanpath comparison that compared both the temporal and the spatial information of fixations. In *string-edit* approaches, gaze sequences are converted into strings of letters, where each fixation of a different AOI is assigned a specific alphabetical character (Anderson, Anderson, Kingstone, & Bischof, 2015a). Furthermore, additional information about the length of the fixation can be included by repeating a letter based on the fixation duration (Cristino et al., 2010). Finally, by counting the number of operations needed to convert one sequence into the other, by using for example the Levenshtein distance (Levenshtein, 1966), a score is calculated to assess the similarity between eye movements in the context of a task (Foulsham et al., 2012).

One algorithm that was successfully adapted from computational biology to eye movement analysis is the Needleman-Wunsch algorithm (Kübler et al., 2017). Compared to the traditional *string-edit* approach, this algorithm allows local alignments between matching AOI patterns of two scanpath sequences.

In over two decades, various algorithms have been proposed to further improve gaze sequence comparisons, such as MultiMatch (Dewhurst et al., 2012), SubsMatch 2.0 (Kübler et al., 2017), EyeMSA (Burch et al., 2018) or ScanMatch (Cristino et al., 2010). MultiMatch compares the similarity of scanpaths as geometric vectors, including measures of saccade length and direction, without needing to couple ET data to predefined AOIs (Dewhurst et al., 2012; Jarodzka et al., 2010). SubsMatch 2.0 classifies eye movements between groups based on *k*-mer subsequences, while EyeMSA allows pairwise and multiple sequence alignments. For an in-depth description see Nicola C. Anderson’s et al. (2015a, b) review on scanpath comparison methods.

In the context of expertise development, the majority of ET studies have applied gaze sequence similarity in the following two ways: the evaluation of experience-related eye movement similarities and the classification of the expertise level based on the gaze sequence. McIntyre and Foulsham (2018b) have successfully shown that the gaze sequences of subjects, within the same level of expertise, are more similar than between subjects of different expertise groups. Castner et al. (2020) proposed a model for scanpath classification that is capable of extracting expertise-specific gaze behavior during a study of dental radiograph inspection.

However, as previously mentioned in the introduction, these approaches have some known limitations. One of the biggest limitations, next to the high computational cost of pairwise sequence comparison, is that similarity calculation is an essentially reductionist approach that reduces gaze behavior to a single cumulative score. While many measures of similarity can be used as a metric to determine behavioral differences between groups (Fahimi & Bruce, 2020), it does not allow one to infer which gaze sequences are developed during the evolution of novices to experts. Measuring similarity over time would indicate that individuals behave more similarly to experts, but the question would remain as to which of the gaze sequences changed during training.

Therefore, a metric is sought that, firstly, keeps the contextual temporal and spatial information of a specific task or domain, while, secondly, allows quantitative measurement of gaze patterns and, thirdly, enables one to infer the level of expertise development. Here, we propose to apply *k*-mer analysis object, or AOI, related gaze sequence patterns.

### *k*-mers

In the field of computational biology, it is a common approach to identify similarity relationships between DNA sequences, with the goal to gain a fundamental understanding of how biological organisms function (Liu et al., 2013). Next to the Needleman–Wunsch algorithm, *k*-mer analysis has established itself as a simple but effective sequence analysis method (Ren et al., 2017). Compared to sequence alignment and string-edit approaches, the *k*-mer method segments each sequence into subsequences of length *k* and counts their appearance within the sequence. Hence, sequences can be compared based on the *k*-mer count of each pattern, while the individual components that are contained within each subsequence are conserved. In DNA analysis, each DNA sequence is regarded as a string with four letters (A, G, C, T), and the choice of *k* determines the number of possible combinations, with *no. of combinations*=*no*. *of AOIs*^*k*^ (Manekar & Sathe, 2018). Because *k*-mers can be applied to all sequences in character representation, they can be applied to gaze sequences in the commonly used string-edit form. Bulling et al. (2013) have applied *k*-mers to electrooculography (EOG) signals to recognize high-level contextual cues, and Elbattah et al. (2020) have used *k*-mers to describe sequence patterns of fixations and saccades to assist the automated diagnosis of autism.

In the present study, we have used higher-level ET data that was created using fixation-to-object mapping. Each dwell on an AOI was assigned a specific letter. Consequently, each *k*-mer pattern both preserves the sequence of *k* successive looked-at AOIs and allows us to compare different expertise levels by evaluating the appearance count of frequently appearing patterns.

## Method

### Subjects

Thirty subjects, mostly students, participated in the study (16 male, 14 female, mean age ± SD = 23.76 ± 2.3 years). All participants reported normal or corrected-to-normal vision and no neurological conditions. Each participant provided informed consent prior to testing and received no monetary compensation.

### Stimuli

As was mentioned in the introduction, it is desirable to record data of the same novices over multiple trials, in order to observe if the same behavioral patterns that are found in experts are developed with increasing experience. Moreover, the comparison of these behaviors across different experience levels will verify if specific tasks induce idiosyncratic gaze sequences. However, in most ET studies involving medical expertise, the investigated task can take years to master, which makes repeated data recording during the stages of development costly and, often due to time constraints of experts, unfeasible. Therefore, it was important to find a suitably simple stimulus which allows the training of task-native subjects and where the development of expert behaviors should be observable within few repetitions while being cognitively demanding. Additionally, the learning complexity should be easily adjustable. We decided to use the assembly of an aircraft model, consisting of Lego-like building blocks, as the study stimulus. In a study on learning curves, Robbins (2019) verified the suitability of such a building block assembly stimulus to portray typical learning behavior over eight repetitions.

**Fig. 1:**
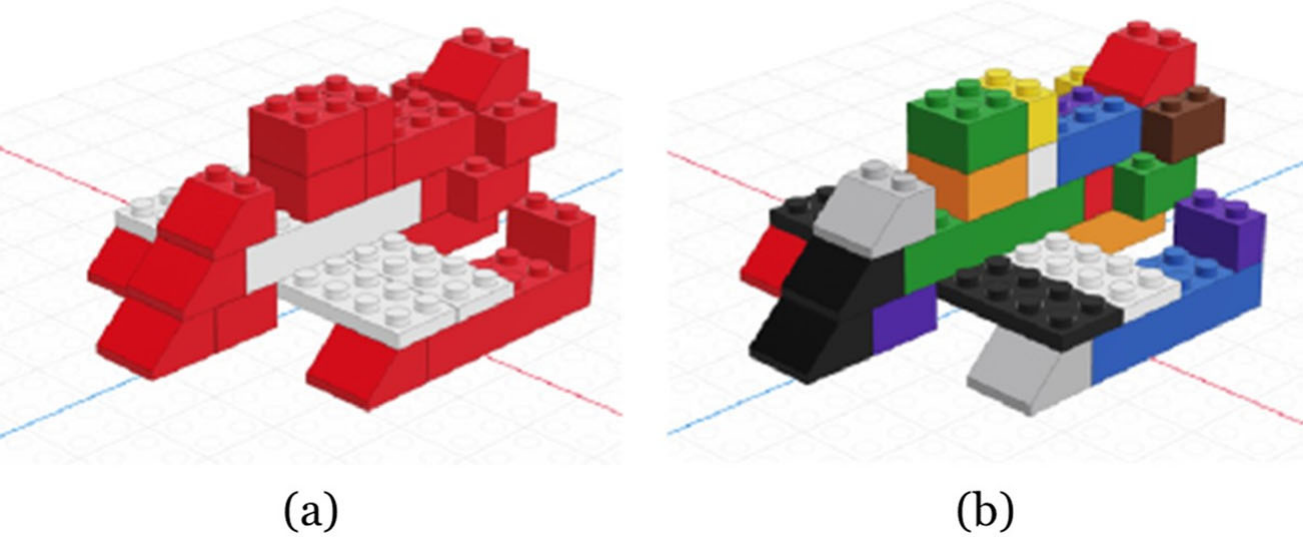
Two building block aircraft models were chosen as assembly stimuli for ***a*** group NOV BC and ***b*** group NOV MC*.* Stimuli differed in the color combination of the model and the number of blocks to choose from

**Fig. 2:**
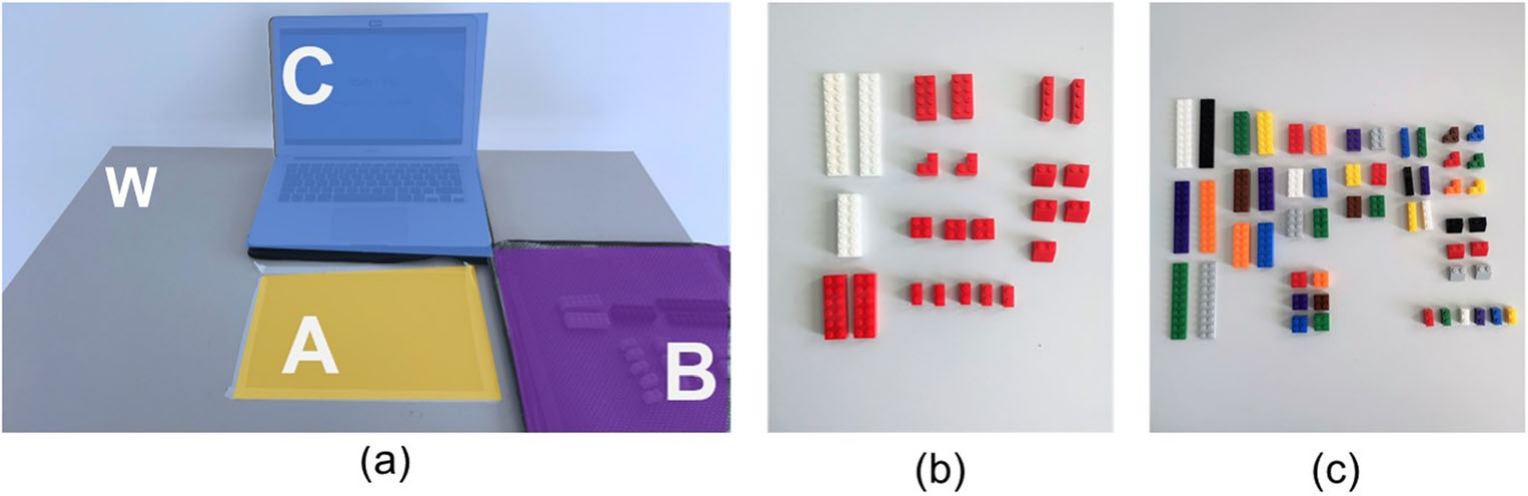
Study setup with the AOIs chosen for data analysis (***a***) and placement of the building blocks for groups NOV BC (***b***) and NOV MC (***c***) at the start of each assembly. Fixations were assigned to AOIs (A) building area, (B) bricks, (C) manual, or (W) white space

To show that the proposed method produces comparable results for different complexity levels, ET data of two stimuli were recorded. Subjects were assigned to either the easier bicolor (NOV BC) or the more complex multicolor (NOV MC) stimulus. Figure 1 shows the aircraft model stimuli for each group. Figure 2a shows the study setup, with three separate task-relevant areas: In front of the subject, a building area was separated by duct tape (A), a tray (B) containing the building blocks was placed to the right of the building area and a digital assembly manual with step-by-step guidance (C) was placed behind the building area. Subjects of group NOV BC were provided with 27 blocks in two different colors (see Fig. 2b). To add the complexity, subjects of group NOV MC were instead given 54 blocks in ten different colors, of which 27 were used as distracting elements (see Fig. 2c).

### Study setup

The study was conducted by dividing the subjects into two groups of 15 participants. Each group was given either the simple bicolor (*n* = 15) or the more complex multicolor stimulus (*n* = 15). Due to the absence of task-native experts, one subject of each group was trained to expert level as a reference for each stimulus (group EXP). The expert training was conducted one day before data recording and was carried out until subjects acquired the ability to finish the assembly repeatedly, without the use of the manual and without making any mistakes. All 30 participants, 28 novices and 2 experts, completed a set of eight successive trials, resulting in a total of 240 recorded assemblies with 240 individual gaze sequences. Each set of trials took approximately 15–25 min to complete (including instructions). The use of the manual was not strictly required; however, all subjects were instructed to respect the building sequence in the correct and color-sensitive order. The arrangement of building blocks in each group was consistent throughout all trials and, after the last building step, a new set of building blocks was provided for the next assembly.

### Recording equipment

Eye tracking data were recorded using the SMI Eye Tracking Glasses 2 and evaluated using SMI’s BeGaze version 3.6 software (SensoMotoric Instruments, Teltow, Germany). The mobile ET glasses record data at a sampling rate of 60 Hz and have a reported gaze position accuracy of 0.5°. The integrated scene camera was recorded with a resolution of 1280 × 960 pixels at 30 frames per second. The audio was simultaneously recorded with the integrated microphone. Before the start of recording, each subject completed a three-point calibration process. The eye tracking ratio (tracking accuracy) was 97.8 ± 1.2% averaged over all participants. No participant data had to be excluded from analysis due to insufficient data quality. We conducted the calibration using the SMI recording unit and a three-point calibration, where the wearer was asked to fixate on three specific markers (top-left corner, top-right corner, and the middle of the bottom edge). During each marker fixation, the experimenter manually confirmed these marker locations on a live-view of the scene camera on the recording device. Afterward, the experimenter made sure that both eyes were clearly visible on the eye camera recordings and calibration was validated using a three-point validation of specific points within the task environment. If the calibration accuracy was not sufficient, calibration and validation were repeated.

### *k*-mer sub-pattern analysis

**Fig. 3:**
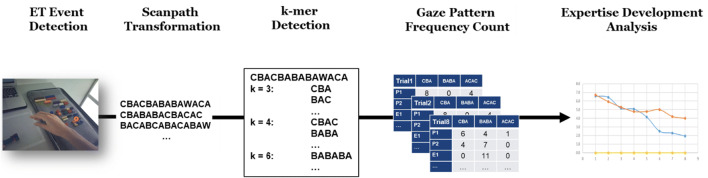
Schematic of the *k-mer* analysis approach

Figure 3 shows the analysis approach for the extraction of *k*-mer gaze sequence patterns. As is the usual practice in ET analysis, the first step is to detect eye movement “events,” particularly eye fixations, defined as a moment in time where the eyes are relatively still at a given point of the visual field. For the fixation detection, we used SMI’s event detector algorithm with default settings (required fixation duration of 50 ms and a peak saccade velocity of 40°/s). Thus, we obtained a sequence of eye fixations for each recording, each fixation characterized by its starting time and duration. We then defined four task-related objects as AOIs for semantic mapping of each fixation. Semantic gaze mapping was conducted manually. Figure 2 shows the AOIs defined for data analysis, namely *building area* (A), *bricks* (B), and *manual* (C). All fixations that did not fall into these AOIs were assigned to white space (W). Using these specific letters, the gaze sequences of each task were converted into *string-edit* representation. Here, we chose the collapsed form of string representation, where successive fixations on the same AOI, also called dwell, are collapsed into a single letter. Consequently, the number of occurrences for each *k*-mer of length *k* = 1 is equal to the AOI dwell count, whereas the occurrence of each *k*-mer of length *k* = 2 is equal to the number of AOI transitions between two AOIs. Consequently, the results for *k* = 1 and *k* = 2 were evaluated as traditional AOI metrics *dwell count* and *AOI transition*, along with the average *fixation duration* and *dwell time*.

The *k*-mer sub-pattern detection was carried out using MATLAB R2019a. For each scanpath, we applied MATLAB’s *nmercount* function to retrieve all *k*-mer patterns of length *k* = {1, 2, 3, 4} in string vector form. Next, we applied the *count* function, with the *k*-mer vector entries as input, to count the appearance frequencies of each pattern. The *count* function was used to count the unique appearances of sequences. The appearance frequency was calculated in each of the eight consecutive trials, allowing the analysis of how these gaze sequences change while novice subjects develop expertise in the given task.

Furthermore, we investigated the specificity of gaze sequences concerning a certain level of expertise by determining the variety of *k*-mers that appeared throughout all trials, separately for experts and novices. Here, we calculated the average relative frequency of each pattern over all trials, for the analytical evaluation of predominant gaze patterns. The equation used is shown in Eq. 1.

1 $$ Relative\ frequency\ of\ {Pattern}_i=\frac{Frequency\ of\ {Pattern}_i}{\sum \limits_{i=1}^{Number\ of\ \mathrm{k}-\mathrm{mers}} Frequency\ of\ {Pattern}_i} $$

The relative frequency of a pattern was calculated by dividing the average appearance frequency of each pattern by the sum of all pattern frequencies and expresses how dominant a pattern was in the overall gaze sequences. For our analysis, only patterns with a relative frequency of more than 1%, were deemed to be relevant gaze patterns and further considered for the analysis of the development throughout the trials.

### Statistical analysis

For the statistical analysis, novice subjects were separated into two groups based on the complexity of their stimulus, group NOV BC or NOV MC. Expert subjects were not considered for statistical analysis due to the lack of significant participant numbers. The statistical analysis of the sequential gaze patterns was conducted using IBM SPSS Statistics 26 and RStudio 3.6.2. First, the data were tested for equal variances using Levene’s test of equal variances, with *p* > 0.05 as the threshold. Subsequently, changes in the appearance frequency of gaze patterns throughout an increasing number of trials were tested within groups using *t*-statistics of dependent variables. To test whether the stimulus complexity induced differences in pattern slopes and intercepts, an analysis of variance (ANOVA) of the aggregated summary statistics was applied between NOV BC and NOV MC. Due to the violation of normal distribution of data and the inequality of variances of some *k*-mer patterns, we used linear regression to estimate the slope and centered intercepts of the pattern frequencies of each participant. Differences in the slope between groups indicate different learning rates of *k*-mer patterns, while different intercepts indicate different pattern occurrences. The data were analyzed statistically, with an alpha of α = 0.05. Additionally, summary statistics were calculated to investigate the change in pattern frequencies of each expertise group.

## Results

Table 1 shows the measured mean *trial times* and observed *use errors* during the assembly for all participant groups. Novices that learn on a more complex stimulus took significantly longer to complete the task (*p* = 0.044), but show a similar decline in the *trial time*s with repeated assemblies (*p* = 0.871). The overall *use error* rate was zero for experts and below 1 for all novices.

**Table 1:** Results of the task completion time and the use errors

	Trial							
	1	2	3	4	5	6	7	8
EXP								
Task completion time [s]	111.8 ± 39.3	92.6 ± 35.3	83.7 ± 27.7	85.7 ± 30.8	77.8 ± 21.3	83.4 ± 25.3	79.0 ± 24.4	84.2 ± 22.1
Use errors [-]	0.0 ± 0.0	0.0 ± 0.0	0.0 ± 0.0	0.0 ± 0.0	0.0 ± 0.0	0.0 ± 0.0	0.0 ± 0.0	0.0 ± 0.0
NOV BC								
Task completion time [s]	159.9 ± 42.8	110.4 ± 21.6	104.1 ± 23.5	98.3 ± 19.5	93.8 ± 14.5	89.7 ± 16.2	89.6 ± 24.4	85.9 ± 23.2
Use Errors [-]	0.4 ± 0.8	0.4 ± 0.6	0.6 ± 1.1	0.1 ± 0.4	0.2 ± 0.6	0.2 ± 0.4	0.2 ± 0.6	0.2 ± 0.4
NOV MC								
Task completion time [s]	178.3 ± 40.8	131.2 ± 26.2	118.4 ± 22.6	113.2 ± 16.3	111.6 ± 17.1	108.9 ± 16.6	102.3 ± 20.3	104.8 ± 20.9
Use errors [-]	0.4 ± 0.8	0.4 ± 1.1	0 ± 0	0.2 ± 0.4	0.1 ± 0.4	0.3 ± 0.5	0.1 ± 0.4	0.1 ± 0.4

### Conventional AOI evaluation metrics

Table 2 shows the mean *fixation duration* along with the conventional AOI evaluation metrics *dwell time*, *dwell count* (*k* = 1), and *AOI transition* (*k* = 2). While the *fixation duration* seems to increase linearly for all participants (slope = 5.918, *p* = 0.95), the standard deviation is shown to be in a similar order of magnitude, which indicates a high variation between individual participants in each group. The *dwell count* (*k* = 1), which is the number of gazes per AOI per task, changes similarly for novice subjects. This trend was shown for AOIs *building area* (*p* = 0.081) and *manual* (*p* = 0.236), regardless of whether the task was learned on the simpler or the more complex stimulus, but showed significant differences for AOI *bricks* (*p* = 0.034). Over time, novices look less at the *manual* (slope(C) = −4.287), while the number of times they look at AOIs *building area* (slope(A) = −2.132) and *bricks* (slope(B) = −0.489) reaches a nearly constant range after the third trial (see Table 2). Conversely, experts showed little change in the *dwell count* for any of the three AOIs. A similar trend can be observed in the *dwell time* on AOIs *building area*, *bricks*, and *manual.*

**Table 2:** Averages and standard deviations for traditional eye tracking metrics fixation duration, dwell time, dwell count (*k*-mers of k = 1), and AOI transitions (*k*-mers of k = 2)

ET Metric	*k*-mer	Trial							
		1	2	3	4	5	6	7	8
EXP									
Fixation duration [ms]		320.4 ± 271.2	344.1 ± 286.7	337.2 ± 293.1	325.0 ± 253.9	340.7 ± 301.0	361.5 ± 322.3	320.4 ± 271.2	320.4 ± 271.2
Dwell time [s]	A	41.6 ± 7.0	40.2 ± 8.2	33.7 ± 4.6	35.8 ± 6.4	35.0 ± 5.0	35.1 ± 4.9	33.8 ± 3.4	39.3 ± 3.7
	B	21.0 ± 4.7	18.2 ± 6.5	15.8 ± 4.1	16.4 ± 6.4	17.6 ± 6.3	17.5 ± 8.1	15.8 ± 6.3	18.5 ± 7.1
	C	5.4 ± 4.2	4.9 ± 1.4	5.8 ± 0.6	6.4 ± 0.5	8.0 ± 1.9	8.4 ± 0.2	6.1 ± 3.1	6.5 ± 0.8
Dwell count (k = 1) [-]	A	24.0 ± 4.0	20.5 ± 0.5	21.5 ± 2.5	21.5 ± 1.5	21.0 ± 1.0	23.0 ± 3.0	22.5 ± 2.5	22.5 ± 0.5
	B	23.5 ± 3.5	20.5 ± 0.5	21.0 ± 1.5	21.5 ± 1.0	21.0 ± 2.0	22.0 ± 2.0	22.0 ± 2.0	23.5 ± 0.5
	C	5.0 ± 3.0	2.5 ± 0.5	2.5 ± 1.5	3.0 ± 0.0	2.0 ± 0.5	3.5 ± 0.5	2.5 ± 0.5	1.0 ± 0.0
AOI Transitions (k = 2) [-]	BA	22.5 ± 2.5	20.5 ± 0.5	21.0 ± 2.0	21.5 ± 1.5	21.0 ± 1.0	22.0 ± 2.0	22.0 ± 2.0	22.5 ± 0.5
	AC	2.5 ± 1.5	1.5 ± 0.5	1.5 ± 0.5	1.5 ± 0.5	1.0 ± 0.0	2.0 ± 1.0	1.5 ± 0.5	1.0 ± 0.0
	CA	1.5 ± 1.5	0.0 ± 0.0	0.5 ± 0.5	0.0 ± 0.0	0.0 ± 0.0	1.0 ± 1.0	0.5 ± 0.5	0.0 ± 0.0
	CB	1.5 ± 1.5	0.5 ± 0.5	0.0 ± 0.0	1.0 ± 1.0	0.0 ± 0.0	0.5 ± 0.5	0.0 ± 0.5	0.0 ± 0.0
NOV BC									
Fixation duration [ms]		332.9 ± 316.2	329.2 ± 314.2	332.3 ± 342.7	348.5 ± 339.9	349.7 ± 334.0	353.3 ± 341.1	359.4 ± 348.9	370.0 ± 340.2
Dwell time [s]	A	70.0 ± 23.4	53.1 ± 14.1	54.5 ± 17.3	52.3 ± 14.2	48.6 ± 9.5	47.7 ± 12.2	48.8 ± 14.6	47.4 ± 17.0
	B	13.5 ± 3.4	13.5 ± 3.3	14.3 ± 4.0	14.7 ± 5.2	14.7 ± 4.7	14.4 ± 3.9	15.6 ± 6.2	14.8 ± 5.2
	C	50.6 ± 19.1	26.4 ± 7.6	22.0 ± 5.7	18.6 ± 3.2	16.4 ± 2.9	13.8 ± 5.3	12.3 ± 5.2	11.9 ± 5.3
Dwell count (k = 1) [-]	A	35.4 ± 10.5	23.3 ± 4.3	22.3 ± 5.5	20.4 ± 3.5	19.9 ± 3.3	19.8 ± 2.8	19.6 ± 3.5	19.4 ± 4.2
	B	21.6 ± 3.4	19.2 ± 3.2	19.9 ± 4.5	19.0 ± 2.7	18.9 ± 2.8	18.6 ± 2.8	19.3 ± 2.8	19.3 ± 3.3
	C	42.6 ± 11.7	28.4 ± 5.3	23.8 ± 7.2	19.2 ± 4.9	18.3 ± 6.0	14.9 ± 7.2	12.9 ± 7.8	10.7 ± 7.1
AOI Transitions (k = 2) [-]	BA	12.3 ± 5.0	12.6 ± 4.8	13.6 ± 3.8	14.9 ± 3.2	14.4 ± 3.9	15.0 ± 4.3	15.8 ± 4.1	16.0 ± 4.7
	AC	31.1 ± 10.6	19.8 ± 4.1	16.4 ± 4.7	13.6 ± 4.7	12.2 ± 4.4	9.8 ± 4.5	8.6 ± 5.3	7.6 ± 5.2
	CA	23.1 ± 11.4	10.7 ± 5.2	8.6 ± 5.0	5.4 ± 2.4	5.5 ± 2.3	4.7 ± 3.1	3.7 ± 3.5	3.4 ± 3.0
	CB	17.6 ± 1.4	16.0 ± 2.1	13.9 ± 3.2	12.1 ± 4.1	11.1 ± 4.3	8.5 ± 4.5	7.5 ± 4.9	6.3 ± 2.0
NOV MC									
Fixation duration [ms]		287.8 ± 274.3	295.2 ± 278.0	299.3 ± 276.4	311.0 ± 310.2	312.9 ± 306.6	319.7 ± 313.0	320.6 ± 307.4	325.3 ± 311.9
Dwell time [s]	A	70.8 ± 23.3	63.8 ± 22.3	52.7 ± 13.4	51.8 ± 10.9	50.0 ± 12.6	50.3 ± 11.1	47.8 ± 13.0	50.3 ± 14.2
	B	25.8 ± 4.9	21.5 ± 4.1	20.2 ± 2.2	20.4 ± 4.4	19.5 ± 4.0	19.7 ± 4.7	19.4 ± 3.6	18.6 ± 3.3
	C	52.3 ± 10.9	34.5 ± 18.2	23.4 ± 6.2	21.0 ± 5.6	20.3 ± 5.3	19.5 ± 6.4	16.2 ± 4.9	16.3 ± 5.8
Dwell count (k = 1) [-]	A	42.9 ± 11.3	34.6 ± 17.8	26.9 ± 5.9	26.0 ± 5.6	23.6 ± 4.7	23.6 ± 4.0	21.6 ± 4.5	22.6 ± 4.2
	B	26.5 ± 2.4	23.1 ± 5.4	20.2 ± 2.6	20.7 ± 2.1	19.9 ± 1.6	20.3 ± 2.3	19.4 ± 2.1	20.4 ± 2.6
	C	58.6 ± 12.1	45.6 ± 19.1	34.0 ± 9.2	32.4 ± 10.5	27.9 ± 9.2	27.3 ± 10.8	22.9 ± 10.6	22.4 ± 10.5
AOI Transitions (k = 2) [-]	BA	9.6 ± 4.9	9.7 ± 4.3	10.9 ± 3.8	10.9 ± 4.1	11.7 ± 4.2	12.5 ± 3.8	12.7 ± 4.0	12.9 ± 5.4
	AC	39.9 ± 10.9	30.4 ± 16.1	22.7 ± 6.2	20.1 ± 6.9	18.0 ± 6.5	17.2 ± 7.7	14.7 ± 7.0	15.1 ± 6.7
	CA	33.4 ± 11.6	24.7 ± 16.4	16.0 ± 7.8	15.1 ± 7.8	11.8 ± 6.0	11.0 ± 5.9	8.9 ± 6.1	9.6 ± 5.9
	CB	23.8 ± 3.3	19.5 ± 4.0	16.4 ± 3.5	15.4 ± 3.6	14.3 ± 4.2	14.2 ± 6.2	12.5 ± 6.0	11.9 ± 5.8

AOI building area is transformed to letter A, AOI bricks to letter B, and AOI manual to letter C. Equally, AOI transition bricks – building area is given as BA

For *k* = 2, novices that learned on the simpler stimulus show lower overall pattern frequencies for the transitions *building area – manual* (AC), *manual – building area* (CA), and *bricks – manual* (BC), but higher average numbers for the transition *bricks – building area* (BA), compared to those participants that have learned on the more complex stimulus. With an increasing number of trials, both groups show a tendency to approach the constant number of *AOI transitions* that is observed for the expert group. Notably, only 2-mer pattern BA shows a marginal linear increase for novices in the appearance frequencies throughout eight trials (slope(BA) = 0.532, *p* = 0.937), while the other patterns have a declining tendency for patterns AC (slope(AC) = −3.049, *p* = 0.481), CA (slope(CA) = −2.665, *p* = 0.074), and CB (slope(CB) = −1.576, *p* = 0.663).

### *k*-mer analysis of higher gaze sequences

Next, we analyzed the *k*-mer sequences and the average relative frequency of each pattern for all trials. Here, the data is presented as mean ± standard error of the mean (SEM), unless otherwise noted.

**Fig. 4:**
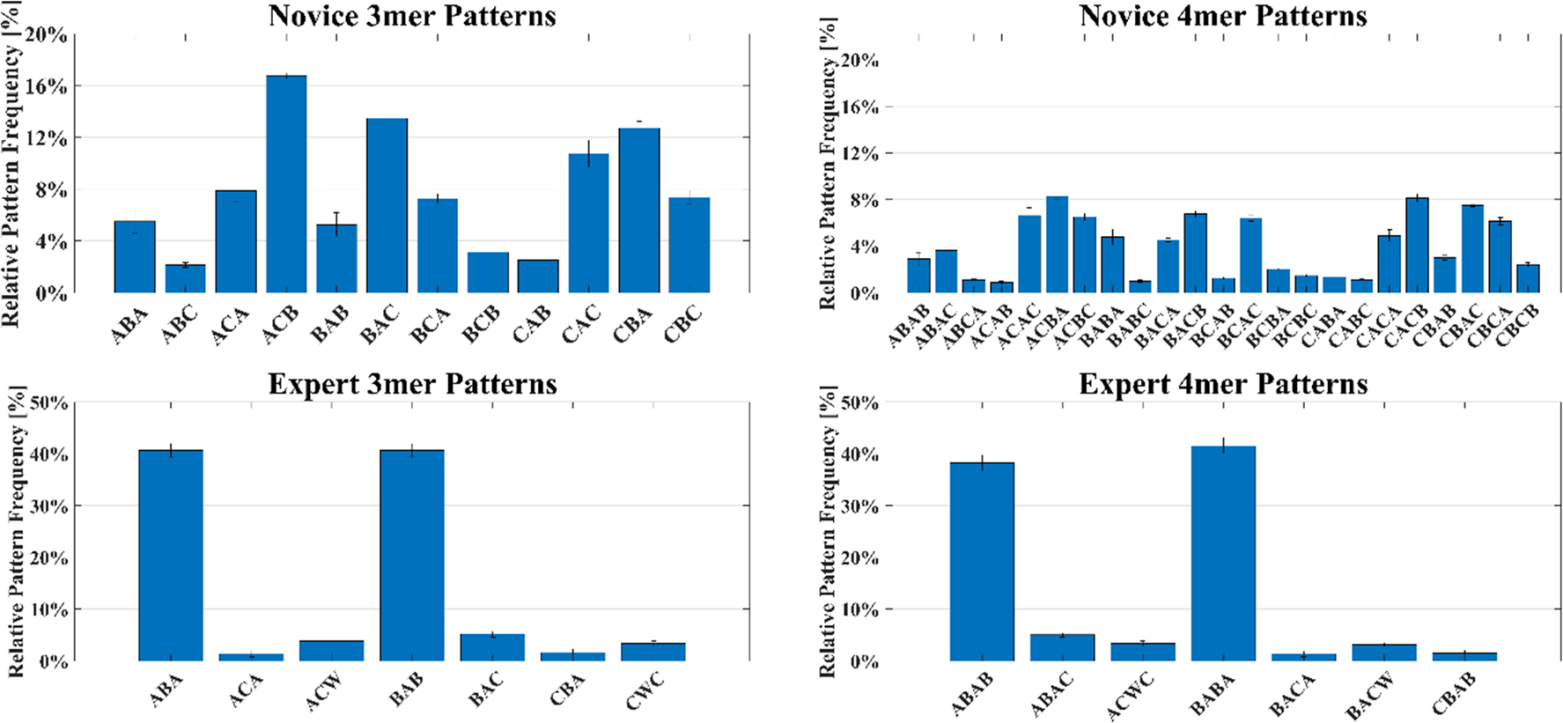
Bar plots displaying *k*-mer patterns with a mean relative appearance frequency of >1% over all trials for *k* = {3, 4}

Figure 4a and b shows the results of the evaluation of the most frequently appearing 3-mer sequences. In the gaze sequences of expert subjects, 7 of 24 possible *k*-mer patterns were measured with a relevant relative appearance frequency. Patterns BAB (40.69 ± 1.12%) and ABA (40.69 ± 1.12%) appeared most frequently. Therefore, more than 80% of the overall expert gaze behavior can be expressed through only two 3-mer patterns. In the gaze sequences of novice subjects, 12 of the 24 sequences appeared with a relevant relative frequency. Patterns ACB (16.76 ± 0.34%), BAC (13.46 ± 0.46%), CBA (12.84 ± 0.52%), and CAC (10.74 ± 1.19%) appeared most frequently. Consequently, more than 60% of the novice gaze behavior can be expressed through the five most frequently appearing 3-mer sequences. Further, more than 90% of the gaze sequence can be explained using the 10 most frequent *k*-mer patterns.

Figure 4c and d shows the results for the analysis of the most frequent 4-mer patterns. In the gaze sequences of expert subjects, again only 7 of 108 possible patterns showed a relevant relative frequency of over 1%. Sequences ABAB (38.24 ± 1.46%) and BABA (41.37 ± 1.42%) appeared to be predominant and showed a strong resemblance to the 3-mer patterns ABA and BAB. Again, approximately 80% of all the visual behavior of experts can be expressed through two *k*-mer gaze sequences. In novice eye movements, the fraction of 4-mer sequence patterns was more broadly distributed, showing 23 of 108 possible pattern appearances. The five most frequent sequences accounted for 37.2% and the 10 most frequent sequences for 65.6% of the gaze sequences of novice subjects.

### Semantic meaning of object-related gaze patterns

**Fig. 5:**
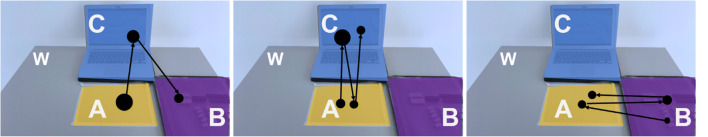
Visualization of the four sequence gaze patterns, ACB (***a***), ACAC (***b***), and BABA and BABABA (***c***) on the study setup

Analyzing the results of conventional ET metrics, we see that experts mainly focused on AOIs *bricks* and *building area*, while the visual attention of novices was shared equally between all three task-relevant AOIs. From the bar plots, it was extracted that the predominant patterns of experts differ from those of the novices. Additionally, the same 3-mer sequence often appears in several 4-mer sequences, i.e. pattern ACB is included in ACBA, BACB, and CACB. Therefore, for the subsequent expertise development analysis, we chose to investigate *k*-mer patterns ACB, ACAC, BABA, and BABABA. Given that with increasing *k* the probability of the appearance of *k*-mer patterns due to chance is reduced, we have included gaze sequence BABABA, a 6-mer extension of BABA, in the analysis. To illustrate the gaze sequence in the study environment, each pattern is depicted as a gaze path in Fig. 5.

By analyzing each *k*-mer sequence pattern along with the ET recordings, we extracted the following semantic meanings: ACB is a triangular pattern between *building area, manual*, and *bricks.* Subjects who repeatedly exhibited this pattern assembled the brick in the area, looked at the assembly manual for the next building step, and finally grabbed the associated brick on the tray. Pattern sequence ACAC was observed in moments when the subject discovered a building error, resulting in multiple glances between the assembled piece in the building area and the assembly manual. Finally, the 4-mer and 6-mer patterns BABA and BABABA represent repeated consecutive gaze transitions between AOIs *bricks* and *building area*. This behavior was shown when subjects became increasingly familiar with the task and were able to carry out several building steps without the use of the manual. Here, participants’ gaze movements were followed by a reach for one building brick and an assembly in the building area.

Consequently, in the subsequent paragraphs, 3-mer pattern ACB will be referenced as *monitor assembly*, 4-mer pattern ACAC as *consult manual*, BABA as *familiarizing steps,* and 6-mer pattern BABABA as *internalized steps.*

### Gaze pattern frequency

**Fig. 6:**
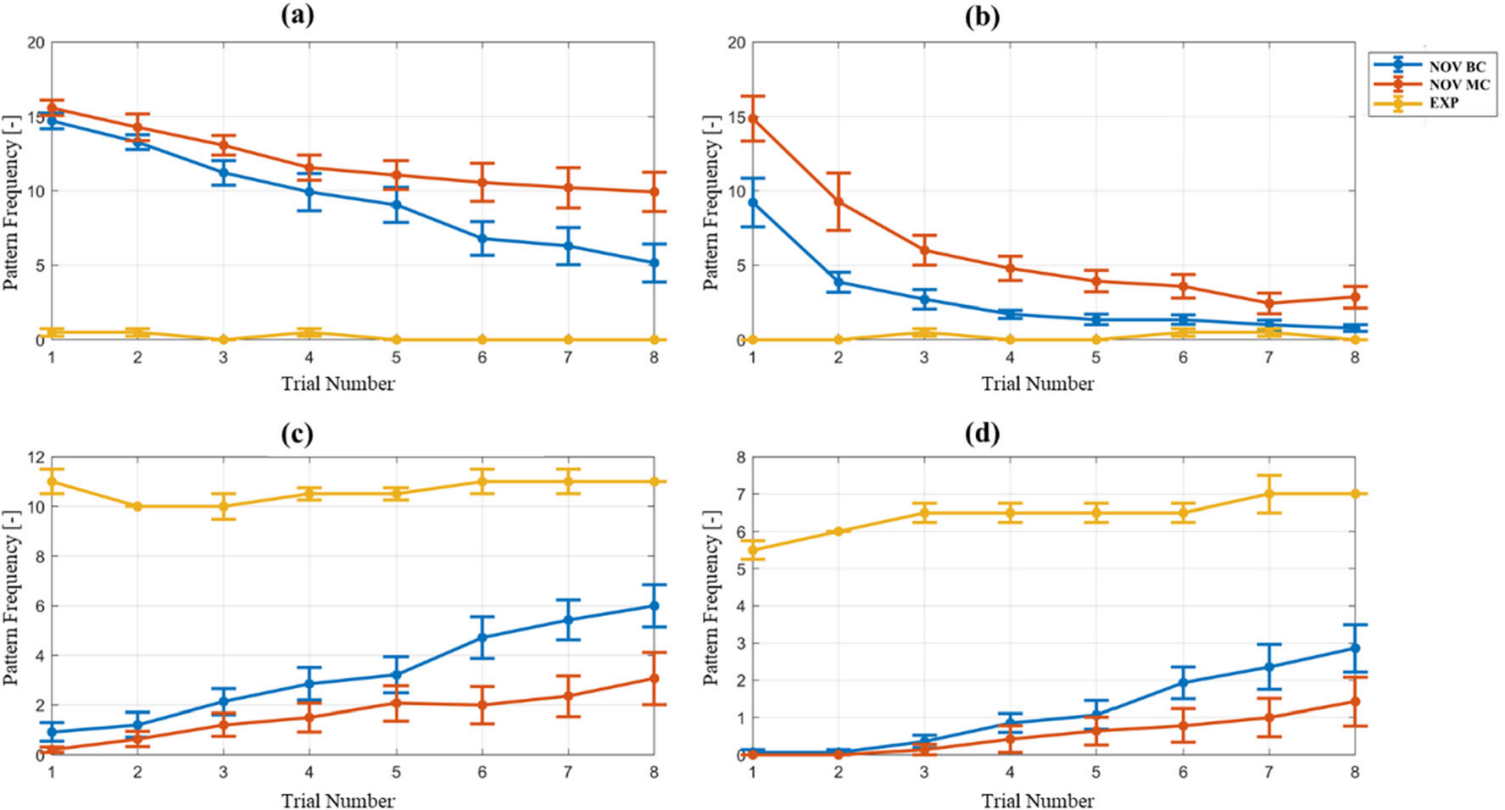
The mean and SEM for patterns ***a*** ACB, ***b*** ACAC, ***c*** BABA, and ***d*** BABABA are shown over eight successive assembly trials

Next, we examined how the frequency of selected larger *k*-mer gaze patterns, i.e. *k* = {3,4,6}, have developed over repeated assemblies. Figure 6 shows the average appearance frequencies per trial for experts and novices. As expected, expert subjects did not show a learning curve for any pattern, but rather constant pattern appearance frequencies. Patterns *monitor assembly* or *consult manual* were absent for most expert trials, whereas patterns *familiarizing steps* and *internalized steps* appeared with constantly high frequencies. For novice subjects, the curve of the pattern *consult manual* resembles that of a traditional learning curve, where the decrease of pattern frequencies between trials becomes smaller with increasing practice. Conversely, the curves of patterns *familiarizing steps* and *internalized steps* indicate linear growth, while the curve of pattern *monitor assembly* indicates linear decline. Notably, the frequency of each of the four gaze patterns converges towards the expert reference appearance frequency count with increasing task practice.

When comparing the *k*-mer frequencies of *k*s of different sizes, the results for *k* = 1 showed a similar *dwell count* on AOI *bricks* (B) for novice and expert subjects over eight trials. For *k* = 2, a difference in gaze strategies between the two expertise levels, for example for AOI *transition bricks – building area* (BA)*,* becomes more evident. For even larger values of *k,* constant appearances of expert gaze patterns such as 4-mer pattern *familiarizing steps* (BABA) and 6-mer pattern *internalized steps* (BABABA), can be measured less and less frequently during novice trials. As these pattern frequencies are close to zero when novice subjects start their learning progress, but increase over time, learning of this specific expert gaze strategy over time is inferred.

Table 3 shows the mean appearance frequency and SEM, for the first and the last trial, as well as the results of a statistical comparison of the pattern frequencies between the first and last trial, using a *t*-test for dependent variables. All subjects showed highly significant differences for all analyzed *k*-mer gaze patterns. The effect sizes greatly exceed Gignac and Szodorai (2016) reported value of *r* = 0.3 for large effects. Thus, within each novice group, the sequence-pattern-based gaze behavior changes significantly from the first to the last assembly.

**Table 3:** Results of the analysis of mean pattern frequencies between the fir*st* and the eigh*th* trial of patterns ACB, ACAC, BABA, and BABABA

*k*-mer Patterns	Trial 1		Trial 8		*t*(13)	*p*	*r*
	M	SEM	M	SEM			
ACB							
Group NOV BC	14.71	0.52	5.14	1.28	8.143	< 10^−3^**	0.914
Group NOV MC	15.57	0.50	9.93	01.32	4.261	.001*	0.763
Group EXP	0.50	0.25	0.00	0.00	-	-	-
ACAC							
Group NOV BC	9.21	1.63	0.79	0.21	5.282	< 10^−3^**	0.825
Group NOV MC	14.86	1.50	2.86	0.73	9.474	< 10^−3^**	0.934
Group EXP	0.00	0.00	0.00	0.00	-	-	-
BABA							
Group NOV BC	0.93	0.37	6.00	0.85	−6.679	< 10^−3^**	0.879
Group NOV MC	0.21	0.11	3.07	1.05	−2.624	.021*	0.588
Group EXP	11.0	0.50	11.0	0.00	-	-	-
BABABA							
Group NOV BC	0.07	0.07	2.86	0.64	−4.545	.001*	0.783
Group NOV MC	0.00	0.00	1.43	0.65	−2.190	.047*	0.519
Group EXP	5.50	0.25	7.00	0.00	-	-	-

A t-test for paired samples was applied on novice subjects separately for groups NOV BC and NOV MC

***p < 0.001, **p < 0.01, *p < 0.05

### The effect of stimulus complexity on expertise development

**Fig. 7:**
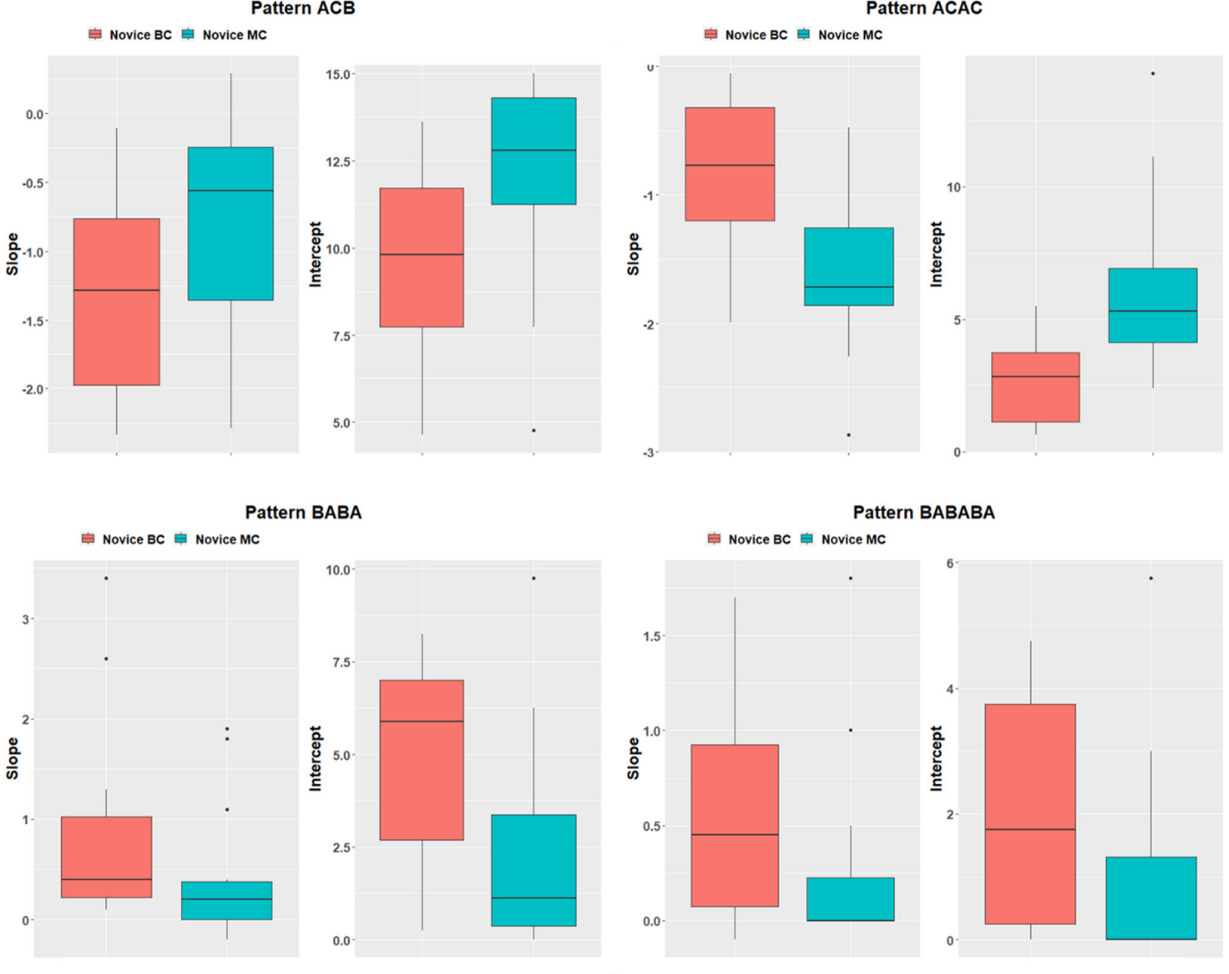
Box plots of the mean slope of pattern frequencies over eight trials. The slopes indicate the rates at which k-mer gaze patterns develop over time, while the intercept show the average occurrence of the pattern in each group

Finally, we investigated the effect of the stimulus complexity on the development rate of perceptual expertise, using a statistical *k*-mer pattern frequency analysis. The slopes and intercepts of the change in the appearance frequencies of the four investigated patterns were compared between novices that learned on the simple (Nov BC) and the more complex (Nov MC) assembly stimulus (see Fig. 7). The null hypothesis considers the slopes of the gaze patterns over time to be parallel, resulting in equal development rates of patterns over time.

The results of the statistical ANOVA test are shown in Table 4. Novices of group NOV BC not only began to focus their attention significantly faster between AOIs *bricks* and *building area* (*p =* 0.0487), using the *k*-mer gaze pattern *familiarizing steps*, but this expert pattern was also observed with a significantly higher appearance frequency (*p* = 0.049). Additionally, learning on a more complex stimulus led to a less significant decrease in gaze behavior *consult manual* (*p* < 0.001), indicating a higher dependence on the task manual during the trials. The decrease in the gaze pattern *monitor assembly* indicates no difference in either stimulus (*p* = 0.543) but shows that a simpler task led to fewer instances where the assembly was monitored (*p* = 0.0374). Similarly, novice participants developed the expert *k*-mer pattern *internalized steps* at the same rate for eight trials (*p* = 0.119) with similar appearance frequencies (*p* = 0.119), regardless of the complexity of the stimulus. Overall, the difference between the complexities of the investigated stimuli did not change the rate at which patterns were developed during the assembly for most patterns, but was shown to influence the average appearance of gaze patterns depending on which stimuli the task was learned on.

**Table 4:** Results of an ANOVA test of the slopes and intercepts between novices groups for the evaluation of the influence of stimulus complexity on gaze pattern development for k-mer patterns ACB, ACAC, BABA, and BABABA, with α = 0.05

Pattern	Global mean	*df*	Sum Sq	Mean Sq	*F* value	*P*
ACB						
Slope	−0.804	1	2.313	2.3130	4.061	.0543
Intercept	8.634	1	43.13	43.13	4.812	.0374*
ACAC						
Slope	−0.321	1	4.626	4.626	13.7	< 10^−3^***
Intercept	2.161	1	86.63	86.63	14.4	< 10^−3^***
BABA						
Slope	0.614	1	.989	.9888	4.277	.0487*
Intercept	3.607	1	17.68	17.681	4.267	.049*
BABABA						
Slope	0.418	1	.347	.3474	2.599	.119
Intercept	1.509	1	2.893	2.893	2.606	.119

***p < 0.001, **p < 0.01, *p < 0.05

## Discussion

In the context of the investigated task, our results for the quantitative analysis of expertise development using the *k*-mer approach led us to the following conclusions:

First, the use of the proposed *k*-mer approach allowed us to investigate the differences in perceptual expertise, by showing that the most common expert and novice fixation sequences differ in their specific AOI composition. By calculating the relative appearance frequency of these patterns, we were able to show that the gaze behavior, in the context of this assembly task, was dominated by only a few *k*-mer patterns. The greater variety of AOI *k*-mer sequences in novice patterns aligns with findings of previous research conducted by Castner et al. (2020), who found that novices experience a larger variety of gaze strategies when compared to experts.

Second, the comparison with traditional AOI evaluation methods showed that the *dwell time* and the *dwell count* (*k* = 1) reveal some learning tendencies regarding the change in novice subjects’ visual attention over time, while the analysis of the *fixation duration* was shown to be inconclusive*.* Learning behavior was shown to be much more evident for patterns of *k* ≥ 2. Through the introduction of a multi-trial study setup, we were able to measure how the gaze behavior of novice participants changed with increasing task familiarity. Compared to studies that have used ET summary statistics as generalizable findings for expert behavior (Jaarsma et al., 2015; Wood et al., 2013), the proposed multi-trial *k*-mer approach could be used as a methodological framework, which reveals those particular gaze strategies that are attributed to a specific level of expertise. Furthermore, our results provide strong evidence that a learning behavior can be measured in the changes in *k*-mer pattern appearances.

Third, the extract gaze behaviors confirmed expected task-related results, showing that experts do not use the manual, while novices learn to use it less over time. Even though the tendency to rely less on the AOI *manual* could be shown in the *dwell time*, the use of larger *k*-mers allowed us to gain additional semantic insights, such as the use of gaze patterns *monitor assembly* and *consult manual* during task-related moments of hesitancy*,* as well as the level of developed task familiarity through the increased use of gaze behaviors *familiarizing steps* and *internalized steps.* Admittedly, it might make little sense to teach students to look less at the manual during training scenarios. However, in a domain such as cardiovascular intervention, where expert surgeons were shown to exhibit fewer gaze transitions than novices between task-related AOIs (Zimmermann et al., 2020), these gaze patterns could be used to assess the current stage of expertise of each subject.

Fourth, using the *k*-mer approach, we were able to quantify both traditional ET metrics (*k*-mer = {1, 2}) and higher-level gaze patterns (*k*-mer = {3, 4, 6}). The consistency of expert pattern appearances resembled a learning plateau, which, as mentioned by Khan et al. (2014), can act as a strong gaze movement-based indicator for expert behavior. Novice subjects showed either an increasing (patterns *familiarizing steps* and *internalized steps*) or decreasing (patterns *monitor assembly* and *consult manual*) pattern development towards the expert plateau. This suggests that the learning of task-specific gaze behaviors can be adequately quantified using the presented method. We were further able to verify previous findings, which demonstrated that gaze behaviors become more similar after a period of learning (van der Gijp et al., 2017), and, using the *k*-mer analysis, specified some of those gaze patterns that were evoked with increased on-task experience.

Finally, the statistical analysis of the pattern slopes suggests that a simpler stimulus reduces the total amount of trials required to reach expert skill levels, while the actual learning rate between tasks was not influenced. This finding should be further investigated in the future using more complex real-world tasks.

We believe that the presented approach adds significant value to the understanding of how expertise related to gaze behavior is developed by contributing advancements to traditional AOI evaluation and string-edit approaches that compare pairwise (Ben Khedher et al., 2017) or group-wise (Burch et al., 2018) similarities of gaze sequences. While the exact AOI composition of the analyzed sequence patterns, i.e. *monitor assembly* or *consult manual*, and their appearance frequencies are expected to differ for each domain of expertise, the *k*-mer approach applies to eye tracking data of all domains.

## Limitations

Limitations of the current work include the limited number of expert subjects within the evaluated ET study. We are aware that the expert data used in this study might not be representative, but the results strongly indicate the consistency of experts’ sequential gaze behavior. Furthermore, this approach suffers from the same limitation as other sequence analysis approaches, which is that the correct or incorrect outcome of a task cannot be directly inferred solely using quantified gaze data and needs to be investigated separately. Some additional uncertainties remain in regard to the optimal choice of *k* and the number of AOIs to be used for the analysis, and should be further investigated. In the future, an AOI frequency-based approach, as introduced by Arzarello et al. (2011), could be implemented to help filter out patterns that are considered identical and thus help automate the choice of analyzed sequence patterns. Furthermore, for mobile ET, conducting semantic gaze mapping manually results in increased manual labor (Vansteenkiste et al., 2015). However, recent advancements in deep convolutional neural network applications have shown that a massive reduction in the effort spent on semantic gaze mapping can be achieved, enabling the automated detection and mapping of looked-at objects (Wolf, Hess, Bachmann, Lohmeyer,, & Meboldt, 2018).

## Conclusion

In the present work, we introduced a novel algorithmic methodological approach for the quantification of expertise development using sequential *k*-mer gaze patterns. Through the evaluation of a simplified natural handling task using a unique multi-trial study design, we contribute to the understanding of the acquisition of task-specific perceptual expertise. By investigating how specific behaviors develop within the same individuals over time, evidence was given that *k*-mer patterns can be a suitable metric to measure and assess learning progress. Additionally, by retaining the object-related AOI identity, gaze sequence patterns are easily interpretable, while containing both temporal and spatial task information. Specifically, for novice education and skill assessment, this approach could provide an answer to the need for a measurement methodology for operator experience and allows us to advance the understanding of task-specific gaze behavior. Consequently, an assessment of the rate with which these patterns approach expert thresholds could serve as a quantitative means to verify the achievement of specific competencies.

By presenting the measurable development of gaze-based expertise using a multi-trial study design coupled with the algorithmic *k*-mer approach, researchers are provided with a promising new methodological framework to study domain-specific expertise and the effects of training on the development of expert gaze strategies.
